# Methods for Untargeted Analysis of Milk Metabolites: Influence of Extraction Method and Optimization of Separation

**DOI:** 10.3390/metabo15090597

**Published:** 2025-09-08

**Authors:** Daisy Wilkie, Brad White, Golnaz Heidari, Rafea Naffa, Gaile Peddie, Gareth J. Rowlands, Paul G. Plieger

**Affiliations:** 1 Fonterra Research and Development Centre, Palmerston North 4472, New Zealand; daisy.wilkie@fonterra.com (D.W.); g.heidari@massey.ac.nz (G.H.); rafea@aidp.com (R.N.); gaile.peddie@fonterra.com (G.P.); gareth.rowlands@fonterra.com (G.J.R.); paul.plieger@fonterra.com (P.G.P.); 2 School of Food Technology and Natural Sciences, Massey University, Palmerston North 4442, New Zealand

**Keywords:** milk, sample preparation, chromatography, metabolomics

## Abstract

Background/Objectives: Cow’s milk is a complex food, and research into its metabolome can provide information useful in the study of animal health, farming practices, food safety and the adulteration of milk. Comparative interlaboratory metabolic analysis is hampered by the lack of standardized methods—a requirement addressed in this study. Methods: We studied the influence of the chromatography column and extraction solvent on the metabolites isolated during untargeted metabolomics. Results: After studying fifteen columns and four extraction solvents, it was determined that an HILIC column offered the best compromise between retention time and separation of metabolites. Each extraction solvent covered a different area of the metabolome, only overlapping with previously annotated compounds. Extraction mixtures containing methanol tend to give better recovery. Conclusions: The choice of extraction solvent was crucial when looking at the difference between samples, but if interest lies only in previously annotated compounds, then there is little difference between the solvents.

## 1. Introduction

Milk is a nutritious food that is important in the growth and development of infants [1]. The high global demand for milk and dairy products has led to increased interest in the quality and safety of dairy-derived food ingredients [2,3]. Milk is a fat-in-water emulsion, comprising water (85–87%), fat (3.8–5.5%), carbohydrates (4.6–5.3%), and proteins (2.9–3.5%), along with minor nutrients such as minerals and vitamins [1,4]. Cow’s milk also contains metabolites in the form of fatty acids, amino acids, vitamins, minerals, organic acids, and carbohydrates. Metabolites are small molecules, normally less than 1.5 kDa, that are formed during cellular activity [5,6]. The analysis of these compounds can deliver insights into fields as diverse as crop science, agriculture, ecology, and medicine [7], as well as providing information about the origins and/or adulterations of milk [8,9].

Metabolomics or metabolomic profiling focuses on identifying and quantifying small molecules, metabolites, in biological systems [10,11]. Metabolites are the end products of cellular regulatory processes, and can provide insights into the response of the biological system to various stimuli [12]. Milk metabolomics is a specific subdiscipline that focuses on the molecules in milk and is a field that is constantly evolving and improving [13,14,15,16]. There are two approaches to metabolomics, targeted metabolomics, which focuses on quantifying a small subset of metabolites using reference standard materials, and untargeted (discovery-based) metabolomics that analyzes as many metabolites as possible without biological bias. Ideally, untargeted metabolomics should cover the whole metabolome, providing data on the relative quantities of each metabolite [17,18,19]. It is a snapshot of a particular biological system at a particular time.

Given the complexity of milk, and its nutritional importance, there is a need to develop reliable methods to obtain a robust global metabolic profile that gives a broad and rich coverage of the metabolome [20]. A typical untargeted metabolomic workflow involves sample collection, extraction, separation, data collection, and data processing and analysis [14,16,21]. Each step should ensure maximum recovery of metabolites while minimizing metabolite derivatization or degradation that could lead to a false picture of the composition [12,17,19,22,23]. However, each step introduces variation and bias to the results [24]. In this paper, we focus on the extraction and separation steps [25], describing research that aims to identify a simple and reproducible method to extract and separate a broad cross section of metabolites from cow’s milk [20].

All extraction methods influence which metabolites are observed. Study of the milk metabolome is demanding due to its complex nature. Both its high lipid concentration and the protein content negatively impact extraction, purification and ionization of metabolites. Various methods ameliorate these issues. When studying the milk metabolome, it is important to minimize the impacts of fatty compounds. These can adversely affect chromatography, changing peak shape and retention time [26,27], and perturb the mass spectrometry signal by disrupting the ionization of compounds, all of which reduces the number of metabolites observed. Defatting, or ‘skimming’, is achieved either by centrifugation or by liquid–liquid extraction. The latter, exemplified by the Folch extraction [28], separates metabolites by polarity and has the disadvantage of partitioning the metabolites between two phases, either doubling the analyses that must be performed or reducing the coverage of the metabolome. Use of organic solvents denatures proteins and aids in their removal as noted below.

Combining centrifugation and single-phase extraction offers a good compromise of simplicity versus recovery and has multiple advantages including the retention of both polar and non-polar metabolites, minimization of handling, and avoids biasing through partitioning effects [20]. Single-phase extraction promotes protein precipitation, and ensures the metabolites are free from protein interference. The choice of solvent is important as it will influence the recovery of metabolites.

Before investigating extraction methods, it was necessary to identify a column for liquid chromatography-mass spectrometry (LC-MS) that showed optimal universal retention of extracted compounds. Correct column choice is important in untargeted metabolomics with column selectivity determining separation and ease of detection [29,30]. Good separation improves the sensitivity of detection and produces better MS data quality as background noise is reduced. However, untargeted metabolomics by its very definition addresses heterogenous collections of molecules with a broad range of size, polarity, and charge, making column selection taxing. Even a cursory inspection of the literature reveals that there is no standardization of the LC column for the untargeted study of milk metabolites, with both reverse phase C18 [31] and hydrophilic interaction liquid chromatography (HILIC) [21] columns being used.

No method can ensure the total recovery of all classes of metabolite and new protocols are required to broaden the snapshot of the metabolome. This study investigated different extraction solvents to optimize the process of obtaining informative data. Ideally, it is desirable to have a simple, yet robust procedure to extract, separate, and analyze milk metabolites that would give wide coverage of the metabolome with minimum handling. Here we screened fifteen columns and studied single solvent extractions, using four different solvent systems that are commonly reported in the literature: acetonitrile (MeCN) [16,28,32,33] or methanol (MeOH) [14,34,35,36], and 1:1 mixtures of acetonitrile:methanol and methyl *tert*-butyl ether (MTBE):methanol [14,37,38,39]. The results indicated that solvent choice is critical to achieving a valid snapshot of the milk metabolome.

## 2. Materials and Methods

### 2.1. LC Column Selection

Before studying the extraction methods, a range of columns were tested for the best universal retention of extracted compounds. An in-house mixture of compounds, that gave a representative cross section of classes of metabolites, was used for column selection. The components of this mixture are shown in Supplementary Materials Table S1. Selection focused on retention time, peak shape, and reproducibility. Satisfactory retention on the column prevents metabolites from being lost in the void volume and maximizes the possibility of separation [40].

Fifteen columns, seven reverse phase columns, and eight HILICs were tested (Supplementary Materials Table S2). The dimensions of each column were sufficiently similar to permit direct comparisons. The standard mixture was run on each C18 column using a flow rate of 0.2 mL/min with the aqueous mobile phase comprising 0.1% formic acid in water and the organic mobile phase being 0.1% formic acid in methanol. Gradient elution started at 10% organic in aqueous solvent and proceeded to 90% over 16 min (0–16 min). It was then held for 4 min (16–20 min) before returning to 10% organic for the rest of the run. The HILIC columns used a flow rate of 0.4 mL/min with 10 mM aqueous ammonium formate with 1.5% formic acid (pH of 2.6) and 100% acetonitrile. The gradient was 90% to 10% acetonitrile from 0 to 10 min, holding at 10% acetonitrile for 10–14 min, and returning to 90% acetonitrile from 14 to 15 min. This was held until the end of the injection at 20 min.

A Waters AQUITY Premier BEH Amide column was used for the rest of the study; its selection is discussed in the following sections.

### 2.2. Collection of Milk Samples

To minimize variation during the study of the different extraction solvents, a 100 mL subsample of a fresh, unopened bottle of commercial milk was taken for analysis.

### 2.3. Extraction of Metabolites

The collected milk was centrifuged at 11,627 rcf for 10 min at 4 °C to defat the milk. A 10 mL aliquot of the supernatant was taken and placed into a separate container and mixed to ensure homogeneity prior to extraction.

Four different extraction solvents, based on literature precedent, were studied [14,16,21,34,41]: Extraction 1 used 100% acetonitrile (LC-MS grade, Thermo Fisher Scientific, San Jose, CA, USA) [16,41]; Extraction 2 used 100% methanol (LC-MS grade, Thermo Fisher Scientific, San Jose, CA, USA) [34]; Extraction 3 used 50:50 acetonitrile:methanol [21]; Extraction 4 used 50:50 methyl *tert*-butyl ether (Sigma-Aldrich, Burlington, MA, USA):methanol [14]. Keeping all extraction solvents at 4 °C, 800 µL of the solvent was added to 200 µL of the sample supernatant, mixed by vortexing, and then centrifuged at 11,337 rcf for 10 min to remove proteins and other large biomolecules. Each extraction was repeated five times to give five replicates per extraction method. A process blank for each solvent was prepared using the same extraction process and LC-MS grade water (Thermo Fisher Scientific, San Jose, CA, USA) in place of the sample. A 100 µL aliquot of each extract was pipetted into vial inserts for metabolomic analysis. A solvent blank, or background, was made from line B [100% acetonitrile (LC-MS grade, Thermo Fisher Scientific, San Jose, CA, USA)] and injected across the sequence to minimize any carry over between runs. A pooled quality control (QC) sample was made by combining 700 µL from each extract, and then 20 µL of the mixture was aliquoted into seven QC vials.

### 2.4. System Suitability and Quality Controls

#### 2.4.1. Data Quality

The first injection was a system suitability blank [42] containing LC-MS grade water. This would identify any compounds within the mobile phases or LC-MS system itself. To mitigate any contamination peaks from the reagents or laboratory consumables used for the extraction process, a process blank was prepared for each extraction performed.

The order of the sequence for analysis on the instrument was created based on the recommendations of Broadhurst et al. [42] to ensure data integrity was maintained. To mitigate any systemic or random errors due to the instability of the LC-MS, five pooled QCs were run at the start of every sequence until a stable baseline was established. To prevent any bias in the metabolite coverage for a single extraction method, extractions using the same solvent were not run consecutively.

#### 2.4.2. Quality Controls

A pooled quality control sample was used for this study using a subsample of each sample extract. This ensured that the quality control was the same matrix as the sample and would contain the same analytes. This is a more accurate quality control than those prepared from ‘close to’ the same matrix and analytes.

The sequence on the instrument had 42 injections that were each 20 min long. Positive and negative polarities were used for obtaining the data. Consequently, the total run time of each sequence was 14 h.

### 2.5. Untargeted Metabolomics Analysis

The untargeted UHPLC-HRMS analysis was performed on an Orbitrap Exploris 240 Spectrometer (Thermo Scientific, Bremen, Germany) coupled to a Vanquish ultra-high-pressure liquid chromatography (UHPLC) pump and equipped with a heated electrospray ionization (HESI)-II probe (Thermo Scientific, San Jose, CA, USA).

Metabolites were separated chromatographically on a Waters AQUITY Premier BEH Amide (150 × 2.1 mm, 1.7 µm) column. The flow rate of 0.2 mL/min was used with an injection volume of 2 µL. Line A contained 10 mM ammonium formate (LC-MS grade, Sigma Aldrich, Burlington, MA, USA) adjusted to pH 2.5 with formic acid (LC-MS grade, Thermo Fisher Scientific). Line B was 100% acetonitrile (LC-MS grade, Thermo Fisher Scientific, San Jose, CA, USA). The gradient used was that of 90% to 10% B from 0 to 10 min, holding at 10% from 10 to 14 min, and then returning to 90% B over 1 min and finally holding at 90% B until the end of the injection at 20 min. Positive and negative ionization modes with a mass resolution of 60,000 at *m/z* 200 were used. Positive ionization used a voltage of 3500 V, and negative polarity used a voltage of 2500 V. The mass range used was 50–750 *m/z* for both. The HESI settings for both MS and MS/MS had the sheath gas at 35 arb (arbitrary units), the auxiliary gas set to 7 arb, and no sweep gas was used. The vaporizer temperature was set to 275 °C and the ion transfer tube was set to 320 °C. Automatic gain control was set to standard for the instrument. All samples were acquired in data-dependent MS/MS (ddMS2) where the top N ions = 5 with an isolation window of 1.5 *m/z*. The top N ions were selected for fragmentation using stepped collision energy of 25, 50 and 100. For ddMS2, the mass resolution was reduced to 15,000 at 200 *m/z* to allow the collection of more MS2 spectra.

To ensure that MS2 data were only obtained for compounds relevant to the study, AcquireX software (part of Xcalibur version 4.6; Thermo Fisher Scientific, San Jose, CA, USA) was used. This software acquires full scan data (MS1) on the extraction blank and then adds all the *m/z* observed above a user-defined threshold to an exclusion list. This exclusion list is then automatically added into the subsequent data-dependent mass spectrometry (DDA) experiments on the samples, ensuring that *m/z* from the blank are not selected for fragmentation. In this way, MS2 coverage for compounds of interest is increased due to lack of competition with process-related/contaminant compounds.

### 2.6. Mass Spectrometry Method

DDA was used to measure the metabolites eluted from the column. It was important to collect the fragmentation pattern of each mass for use in the annotation process. Using the method described above, greater than 90% of the metabolites detected had MS2 spectra.

Prior to data collection, the mass spectrometer was calibrated at both positive and negative modes using Pierce™ calibration solution (Thermo Fisher Scientific, San Jose, CA, USA). The sequence of injections was randomized. The data generated as (.raw) files were checked for consistency and suitability using FreeStyle™ 1.8 SP2 software package from Xcalibur™ Software, and then processed using Compound Discoverer 3.3 SP1 (Thermo Fisher Scientific, San Jose, CA, USA).

### 2.7. Data Processing

The commercial ‘Untargeted Metabolomics’ template was used with the following modifications: Retention Time was used for peak alignment. The mass tolerance of 5 ppm and minimum peak intensity of 10,000 were used for detection and adaptive curve of max 1 min. The signal/noise threshold was 9. Four positive ion adducts [M+H]^+1^, [M+K]^+1^, [M+Na]^+1^, and [M+NH_4_]^+1^, and two negative ion adducts [M–H]^−1^ and [M+Cl]^−1^ were used to extract the features. The retention time window of 0.2 min was used to group the compounds and create the compound table with a Peak Rating Filter function. Finally, Fill Gaps, SERFF QC and Mark Background were used. Annotation was performed using mzCloud and ChemSpider including the Bovine and Human Metabolomics databases with Milk Composition Database.

### 2.8. Data Statistics and Visualization

The processed data were exported as csv files and then imported into RStudio running using R 4.2.2. The ggstatsplot package was used to explore and visualize the data [43]. Finally, the VennDiagram R package (R 4.2.2) was used to determine the overlapping metabolites in the four extractions [44].

## 3. Results

### 3.1. Column Selection

**Figure 1:**
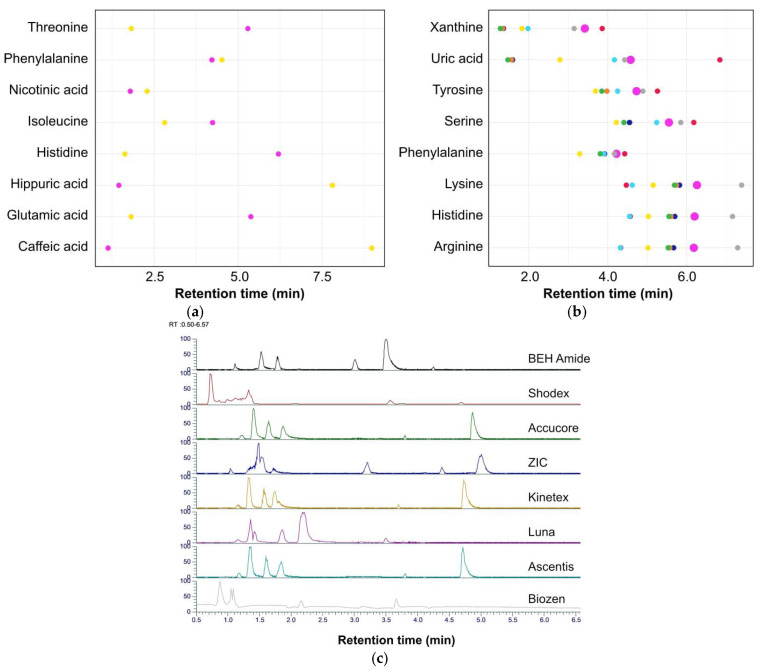
Column selection: (**a**) Comparison of the retention times for eight standards across two columns, including a C18 reverse phase column (Luna C18; ■) and a hydrophilic interaction liquid chromatography (HILIC) column (BEH Amide; ■). (**b**) Plot showing the retention time of the eight standards run on eight HILIC columns (■, Accucore; ■, Ascentis; ■, BEH Amide; ■, Biozen; ■, Kinetex; ■, Luna; ■, Shodex; ■, ZIC). (**c**) Chromatograms showing Total Ion Count for the in-house standard mixture being separated on eight HILIC columns.

For untargeted metabolomics, a good column retains compounds for sufficient time to allow separation but should not result in excessively long run times. Fifteen columns were tested, seven reverse phase columns and eight HILIC (hydrophilic interaction liquid chromatography) columns. For each column, the retention time of an in-house set of standard compounds (Supplementary Materials Table S1) was compared (Figure 1). As expected, the C18 columns showed poor retention for polar compounds with retention times increasing as the compounds became more hydrophobic. Conversely, the HILIC columns retained polar compounds for longer. The eight HILIC columns showed better retention for the range of standard compounds tested than the C18 columns. Peak shape and separation were also important criteria, and the chromatograms for eight of the trialed columns are shown in Figure 1c.

The final selection was a Waters ACQUITY Premier BEH Amide column, which showed the best retention for polar metabolites while also retaining many non-polar metabolites. It displayed better consistency for the compounds; although not always the longest retention time, it was routinely one of the better performing columns.

### 3.2. Quality Control

**Figure 2:**
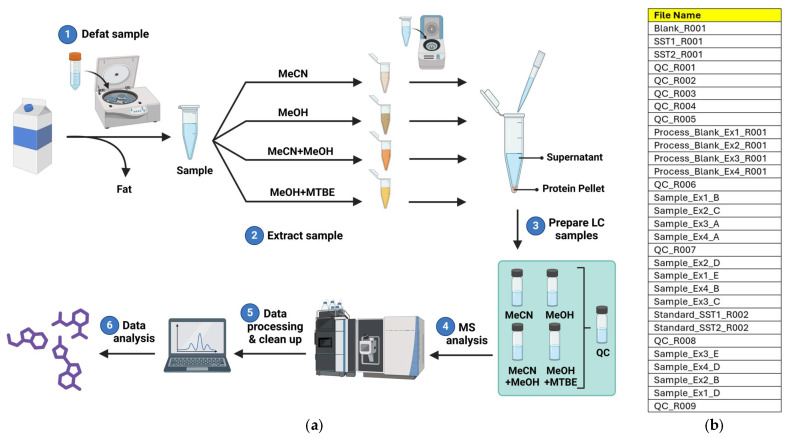
The workflow for the current study. (**a**) A visual description of the workflow: 1. skimming or defatting the milk sample; 2. sample aliquoting and extraction with four different solvent mixtures involving vortexing and centrifugation to remove protein; 3. supernatant for each sample was transferred to sample vials and a pooled quality control created by subsampling each extract; 4. chromatography and untargeted metabolomic analysis; 5. data processing; 6. data analysis. (**b**) The injection sequence was designed to minimize any bias and permit the quality of the data to be ascertained.

The workflow, starting from raw material and proceeding to data analysis, is outlined in Figure 2. Commercial milk was defatted by centrifugation, and then the milk and a water blank were extracted with the four solvent systems. Removal of the precipitated proteins and biopolymers was followed by preparation of the four samples along with a pooled quality control (QC). The latter was injected five times at the start of the sequence to condition both the chromatography column and the mass spectrometer. This ensures stability of the baseline and leads to the collection of more reproducible data. Next, the process blanks were injected followed by the extract samples in a random order as shown in the diagram. The pooled QCs were then injected after every five samples within this sequence. Only the QCs run between the samples were used for the statistical analysis, so that the initial five QCs used to equilibrate the system did not skew the data. Data processing and analysis finished the workflow.

**Figure 3:**
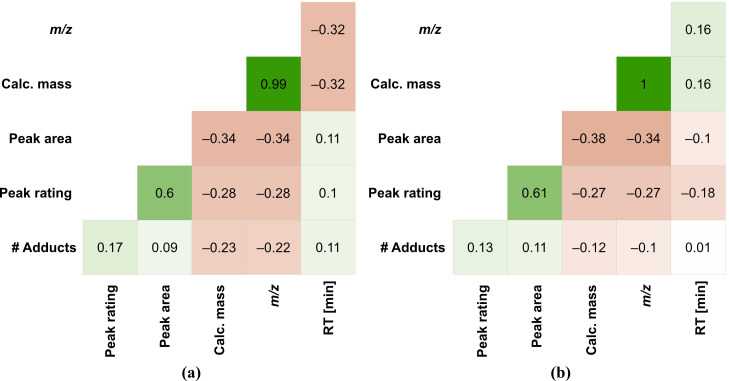
Data quality factors for pooled quality control (QC) sample replicates run on a Waters ACQUITY Premier BEH Amide column with ammonium formate buffer at pH 2.5: (**a**) correlation diagram for the pooled quality control sample replicates (*n* = 17,552) measured in positive ionization mode (Calc. mass = Calculated mass; RT [min] = Retention Time in minutes); (**b**) correlation diagram for the pooled quality control sample replicates (*n* = 47,762) measured in negative ionization mode.

Analysis of the QC indicated that more than 90% of all compounds are in the [M+H]^+1^ or [M–H]^−1^ charge states, a desirable characteristic as it shows that the instrument is free of any adduct-forming contaminants and because these single charge states ionize more reproducibly than other adducts. The high quality of the data, in both positive and negative ionization modes, is highlighted by the analysis shown in Figure 3. The correlation diagrams show the expected relationship between the calculated molecular mass and *m/z* and this supports the observation that most compounds are singly charged with little adduct formation. Similarly, the weak positive correlation between peak rating and peak area is expected. Larger peaks frequently present a better peak shape. It is worth highlighting the lack of strong correlations between peak quality and either retention time or *m/z*. This demonstrates that there is good chromatographic resolution and mass resolution over the length of the run. This validates the quality of the chromatography and justifies the column selection.

**Figure 4:**
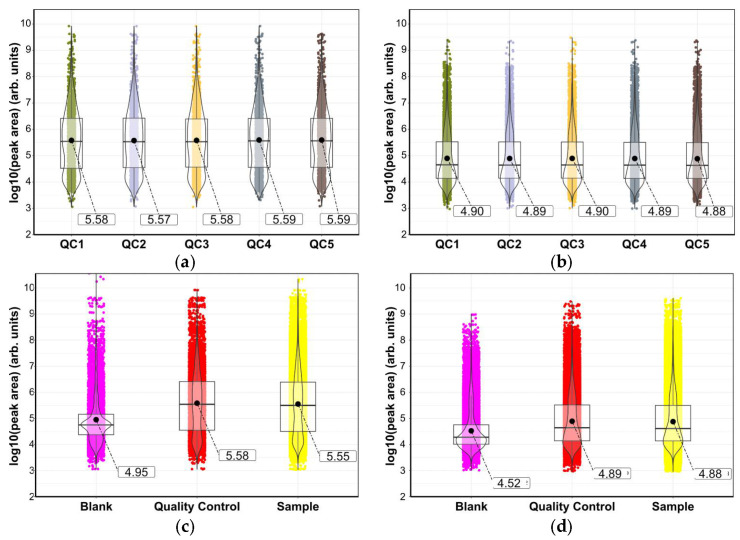
Violin plots comparing the log_10_ peak areas of the pooled quality controls, blanks, and the samples ( ${\hat{\mu }}_{mean}$ values given in boxes). Each plot shows distribution of the log_10_ peak areas for extraction with 100% acetonitrile (MeCN), 100% methanol (MeOH), 1:1 acetonitrile:methanol, and 1:1 methyl *tert*-butyl ether (MTBE):methanol. (**a**) Mass spectra recorded in positive ionization mode before data cleaning (*n* = 9995); (**b**) mass spectra recorded in positive ionization mode after data cleaning (*n* = 5705); (**c**) results from negative ionization mode before cleaning (*n* = 13,820); (**d**) results from negative mode after data cleaning (*n* = 46,590). Samples separated using Waters ACQUITY Premier BEH Amide column with the eluent described in the materials section. Below each violin plot there are tabulated peak distributions; for each extraction method, there is the percentage of peaks with intensities less than 1 × 10^5^, between 1 × 10^5^ and 1 × 10^8^, and greater than 1 × 10^8^. During visualization, some *p*-values were rounded to *p* = 1.00 or *p* = 0.00; these are not true *p* 1 or 0 values, with the latter being *p* < 0.001.

The peak areas for each pooled QC sample were transformed into log_10_ values and visualized in R as a violin plot (Figure 4) [31]. The plot shows that the mean and distribution of the measured metabolites replicate across the five QC samples [43]. This suggests that the mass spectrometry performance over a 14 h run has not changed and has produced high quality data for the samples. The reproducibility of the QC was also replicated in the samples.

A similar comparison of the combined samples with the QC and blanks gave useful information (Figure 4c,d). As expected, the blank has a relatively low mean when compared to the sample and the QC. The low mean results from a higher distribution of low peak areas that is indicative of little impurity or carry-over. The distribution of peaks seen in the violin plots for the QC and sample are similar with both showing a bimodal distribution. This confirms that the QC is an accurate representation of the sample. Results from the negative ionization mode for the sample, QC, and blank show more than four times the number of masses than in positive mode, and these results are skewed towards lower peak areas, as evidenced by the difference in means.

The increase in the mean peak area in the QC and the samples compared to the blank confirms the successful extraction of milk metabolites. The bimodal distribution shown in the violin plots of all QC and samples suggest that a data cleanup process was needed to remove the low area peaks that also have low peak rating or shape. Such processing will produce higher quality data that can permit more reliable comparison between the different extraction solvent mixtures.

### 3.3. Data Processing and Clean-Up

**Figure 5:**
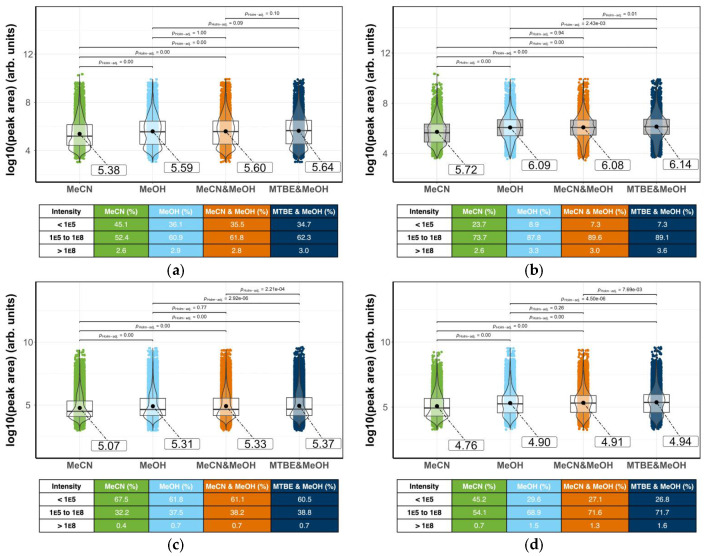
Violin plots showing the results of data processing for the four extraction methods and in both ionization modes ( ${\hat{\mu }}_{mean}$ values given in boxes). The data analysis workflow omits any peaks that fail to meet a minimum threshold; hence, there are no data points below log_10_ = 3. These plots show the variation, or not, in peaks obtained for each type of sample: (**a**) the log_10_ peak areas from the five pooled quality control injections (QC1–QC5) with the mass spectra recorded in positive ionization mode (*n* = 1999). Samples separated using Waters ACQUITY Premier BEH Amide column with ammonium formate buffer at pH 2.5; (**b**) the log_10_ peak areas from the five pooled quality control injections with the mass spectra recorded in negative ionization mode (*n* = 9318); (**c**) the log_10_ peak areas for the blank sample compared with the pooled quality control samples and the sample with the mass spectra recorded in positive ionization mode [*n* = 7996 (blank); *n* = 9995 (QC); *n* = 39,980 (sample)]; (**d**) the log_10_ peak areas for the blank sample compared with the pooled quality control samples and the sample with the mass spectra recorded in negative ionization mode [*n* = 37,272 (blank); *n* = 46,950 (QC); *n* = 186,360 (sample)].

As seen in Figure 5, using the workflow developed with Compound Discoverer (see Section 2.7), a large proportion of features was eliminated in the data cleaning steps. The data processing removed low-quality data points corresponding to features with low area and poor peak shape. Only metabolites observed in both the QC and sample were kept, ensuring confidence and reproducibility in peak area and retention time. Data cleaning removed any compound found in the background that was observed in fewer than three QCs, had a QC relative standard deviation (RSD or coefficient of variation) of >20%, or a group coefficient of variation of >20% for every cell holding a value. The value of 20% for the RSD of the QC was chosen based on Lewis et al. 2016 [34] and Want et al. 2010 [45,46].

Data cleaning resulted in the removal of 3149 of the detected peaks, 31.5%, from the positive mode (down from 9995 before cleaning to 5705), and 32,770 or 70.3% of the peaks removed from the negative mode (46,590 data points down to 13,820). As expected, comparing the plots for pre- and post-data cleaning (Figure 5a versus Figure 5b and Figure 5c versus Figure 5d) indicates that data processing and clean-up predominantly removes peaks with small areas. These tend to be less reproducible peaks that fail to fit quality control requirements or are present in the background samples.

Pair-wise comparisons were performed using Student’s *t*-test between all the extractions to evaluate whether the distribution of peak areas significantly differed. The *p*-value was adjusted using the Holm method, and any result with an adjusted *p*-value of < 0.05 was considered significant (see Figure 5 for values).

Inspection of the positive ionization mode data prior to data cleaning reveals extractions with methanol, acetonitrile:methanol mixture, and the *tert*-butyl methyl ether:methanol mixture, which show similar bimodal distributions of peak areas, with no significant difference between these solvents. There was a significant difference between the distribution of the peak areas from the acetonitrile extraction compared with the other three solvent mixtures. The mean is lower, and the violin plot shows more peaks are skewed to a lower analyte response. This suggests that acetonitrile is less effective at extracting metabolites than the other methods. The acetonitrile:methanol mixture and the methanol extractions are very similar in the distribution of peak areas.

After data cleaning, the methanol and acetonitrile:methanol mixture still had a similar distribution of peak areas compared with each other; however, comparisons of the other extraction solvents showed significant differences. Data cleaning transformed the bimodal distributions for the methanol, acetonitrile:methanol, and *tert*-butyl methyl ether:methanol extractions into a more unimodal pattern. The mean was increased, indicating that the data cleaning removed unreliable peaks with low area values. In contrast to the other solvents, while acetonitrile saw an increase in mean peak area after data cleaning, it did retain a bimodal pattern.

In the negative ionization mode, all extraction solvents gave a lower mean peak area with data before cleaning showing the mean skewed to lower values. After cleaning, there was a bimodal distribution with two approximately equal centers, one close to the mean and one around the lower values.

Post data cleaning, for both ionization modes, there was a significant difference between the peak area distributions of all the extraction solvents, except when comparing the methanol extraction and the acetonitrile:methanol mixture. Acetonitrile gave results different from the other solvents, with an overall lower mean in both modes. This indicates a larger distribution of analytes with lower peak areas, and suggests acetonitrile is less effective at extracting metabolites.

### 3.4. Statistical Analysis

**Figure 6:**
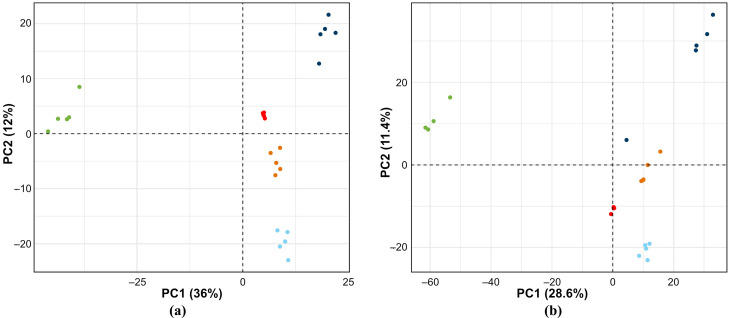
Principal component (PC) analysis of different solvent extractions [(■, acetonitrile; ■, methanol; ■, acetonitrile/methanol (50:50); ■, methyl *tert*-butyl ether/methanol (50:50)] and the QC results (■): (**a**) in positive ionization mode; (**b**) in negative ionization mode. Note that one replicate from the acetonitrile extraction was removed from the PCA for negative mode as it was a clear outlier (>50x removed in both dimensions from other replicates).

Principal components analysis (PCA; Figure 6) shows a clear difference in the peak areas of the detected features across the four different extractions. This shows that they extract different classes/abundances of metabolites. The PC1 dimension accounts for 36% of the variability in positive mode (Figure 6a) and 29% in negative mode (Figure 6b). The acetonitrile extraction separates well from the others in the PC1 dimension. The PC2 dimension separates out the differences between the other three extractions. The clustering of the replicates for each extraction, and the tight clustering of the QC replicates cluster reinforce the robustness of the procedure and that the instrument maintained excellent precision across the entire run.

**Figure 7:**
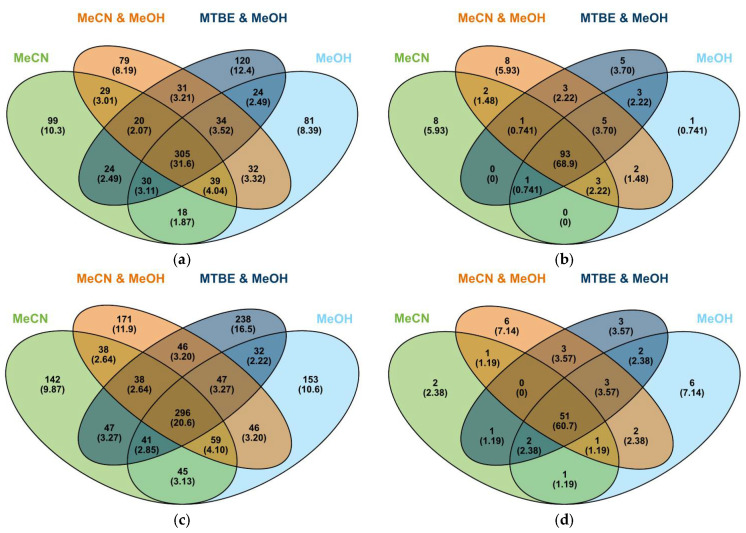
Venn diagrams showing distribution [number and percentage (in parentheses)] of extracted compounds by solvent: (**a**) compounds assigned a molecular formula in each extraction solvent as detected in positive ionization mode; (**b**) annotated compounds detected in positive mode in each extraction; (**c**) compounds assigned a molecular formula in each extraction solvent as detected in negative ionization mode; (**d**) annotated compounds detected in negative mode in each extraction. MeCN, acetonitrile; MeOH, methanol; MTBE, methyl *tert*-butyl ether.

All masses assigned a formula from the four extractions were mapped out and grouped in Figure 7. This shows that there is a large impact of solvent selection on the extraction of metabolites from milk, with only 32% of metabolites being extracted by all four solvents in positive ionization mode, and 21% in negative ionization mode. The number of metabolites extracted by all four solvents increased substantially after the data set was annotated.

Of the compounds assigned a formula, only ones that can be annotated with known milk metabolites may be useful to researchers looking for meaningful differences in milk profiles, unless extensive effort is made to identify the unknowns. For this reason, the chemical formulae found were annotated primarily using the Milk Composition Database [1]. Comparing extractions for the presence of these known metabolites shows a reduced impact of the chosen extraction solvent, with 68.9% in positive ionization mode and 60.7% in negative ionization mode of annotated compounds being extracted by all solvents (Figure 7).

**Figure 8:**
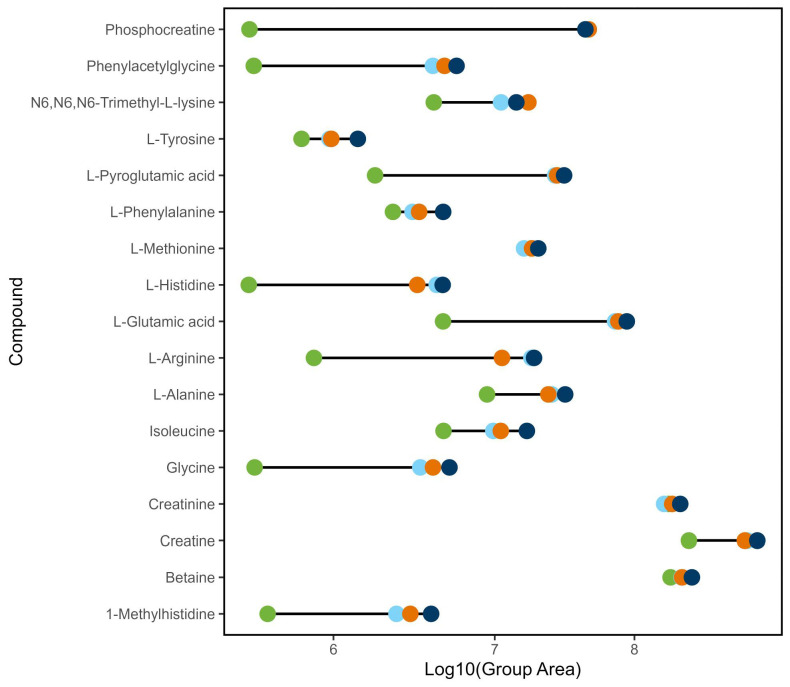
Extraction of amino acids and derivatives in positive ionization mode. The dumbbell plot shows that each solvent extracts differing quantities of the identified metabolites as indicated by the plot of the log sample peak areas: ■, acetonitrile; ■, methanol; ■, acetonitrile:methanol (50:50); ■, methyl *tert*-butyl ether:methanol (50:50).

Various metabolites were then assigned putative annotations by MS2 and MS1 spectral matching to the m/zCloud endogenous metabolites library and the Milk Composition Database [1]. Applying such criteria meant that no annotations were possible for compounds in the negative ionization mode (see Supplementary Materials Tables S3 and S4 for the full list of annotated metabolites for each extraction). The effectiveness of each extraction method for annotated metabolites in positive ionization mode is displayed in Figure 8. This indicates that the different solvent extractions cover different classes of metabolite or show different propensities for each compound depending on their physical properties.

## 4. Discussion

In this study, we examined four solvent extraction systems with the goal of maximizing coverage for the untargeted metabolomics of cow’s milk; missing any metabolite space unconsciously skews the results. Yet simply obtaining large numbers of features is not enough; the data generated should be robust and reproducible so others in a different laboratory can follow the same process and have confidence that the gathered results are comparable. This requires using good practice to generate and validate data, followed by a reliable protocol to ‘clean’ or remove poor quality data [12,17,18,19,47,48]. To allow meaningful comparisons to be made, the same column and mobile phase were used across the study. The ACQUITY BEH Amide column with an ammonium formate buffer/acetonitrile mobile phase gave satisfactory separation for a series of test compounds with various degrees of polarity. Column selection was based on the best retention of metabolites with a broad spectrum of chemistries, along with the most symmetrical and reproducible peak shapes.

The recommendations of Broadhurst et al. [42] were followed for quality assurance and quality control. Prior to batch runs, the system was conditioned by repeated injections of the pooled QC sample to enhance stability and reproducibility. The generation of quality data was further aided by careful ordering of injections in the batch run according to the injection protocol of Broadhurst et al. [42]. Finally, the injection order of the four sample extract injections themselves was randomized. By ensuring there is a quality assurance system in place when performing the study, quality data are produced, and we are confident that this will be reproducible in other laboratories.

An initial examination of the data revealed that over 90% of the features detected in both positive and negative modes were singly charged adducts (Figure 3). This provides superior sensitivity for any given metabolite compared with multiply charged adducts as the latter form when the metabolite fragments into several features of smaller intensity. The correlation diagram in Figure 3 provides greater confidence that we are obtaining good coverage. As expected, *m/z* correlates strongly with the calculated molecular mass. More useful is the absence of a correlation between the retention time and peak area, peak rating, *m/z*, and calculated molecular mass, which indicates that neither the ionization nor retention biased the quality of the peaks. This validates that mobile phase composition and compound co-elution have a minimal effect on the size and quality of individual peaks, which is a key feature of a robust method.

The processed data were visualized as a series of violin plots showing the distribution of the log_10_ peak area of features extracted (Figure 4). The behavior of the pooled sample extract (QC) injections was consistent across the run, giving reassurance of the stability of the platform (Figure 4a,b). Examining the blank, QCs and samples (Figure 4), the features in the blank show a unimodal distribution in both positive and negative modes and are tightly clustered at lower peak areas as would be expected for features that do not correspond to true metabolites. For the QC and sample, the positive mode data are bimodally distributed, with a greater difference in the median area compared to the blank. The distribution of features in the negative ionization mode data is unimodal for all injections. This suggests that there are numerous low mass compounds present in the milk matrix that readily ionize in the negative ionization mode. Interestingly, there are many usable features found in the QCs and samples but absent in the blank in both ionization modes.

Again, Broadhurst et al. [42] formed the basis of our protocol for cleaning the processed data set. The cleaning metric removes features with a relative standard deviation (RSD) greater than 20%, filtering out many features with small peak areas. These, by their very nature, are less likely to be reproducible.

In both ionization modes, the distribution pattern of the features extracted with acetonitrile only is distinctly different from the methanol-containing extraction solvents (where *p* < 0.05). The difference is more pronounced in the positive mode, where the acetonitrile-extracted features skew towards lower peak areas.

For the positive mode, the distribution of peak areas for all extraction solvents was bimodal prior to cleaning. Data cleaning filters many features with small peak areas and the violin plots for extractions with methanol, or methanol mixtures, shifted towards a more unimodal distribution with a higher mean (Figure 5b,d). This emphasizes the superior coverage of the methanol-containing extraction solvents. The acetonitrile remained unimodal, clustered around lower areas. The fact that many of the low abundance peaks met the data cleaning criteria indicates that acetonitrile is extracting numerous compounds in a lower concentration than the other solvent systems. Cleaning enhanced the differences between the median areas of the extraction solvents, albeit slightly.

Compared with positive mode, all extraction solvents gave a lower mean for peak area, both before, and after, data cleaning in negative mode. This suggests that a lower concentration of negatively ionizable compounds was observed and might indicate that the negative mode is less sensitive in this untargeted study. The PCA plots confirm the repeatability of the results with good clustering. They also illustrate the effect of solvent on extraction of metabolites, highlighting the difference between pure acetonitrile and methanol-containing mixtures. The pooled sample extracts (QC) cluster tightly around the origin, as expected. Acetonitrile:methanol also clusters tightly, while MTBE:methanol scatters more than the methanol alone. Methanol, either neat or mixed, affects the data set of metabolites extracted from cow’s milk.

The Venn diagrams (Figure 7) depict the percentage of metabolites extracted by each solvent mixture in both positive and negative ionization modes. There is a striking difference in the distribution of formula-assigned compounds compared to annotated compounds. Choice of solvent is important when inspecting non-annotated, formula assigned compounds but less so for annotated compounds. No solvent extracted greater than 60% of the formula–assigned metabolites in positive mode or 55% in negative mode. Only 21% of these compounds were extracted in all solvents. Inspection of the annotated compounds showed a different pattern. In positive mode 69% of the annotated compounds were common to all solvents, with each solvent extracting more than 80% of the total number of compounds. Results are similar for negative mode with each solvent extracting between 70 and 80% of the compounds and 61% being common to all solvents.

These results show that solvent selection is vital when examining statistical differences between samples rather than attempting to annotate the compounds. The choice of solvent will determine what metabolite space is explored. Annotated compounds appear to be more focused and insensitive to solvent choice. This might reflect the empirical nature of the databases used in the annotation process.

The Venn diagrams do not reveal how efficient each solvent is at extracting each compound (a full list of annotated compounds by extraction mixture is given in the supplementary data). In the Venn diagram for the positive mode, each solvent extracts a similar percentage of molecules, acetonitrile extracts 80% as does methanol while the acetonitrile:methanol mixture extracts 87% and MTBE:methanol extracts 82% of the annotated compounds. Yet the dumbbell plot in Figure 8 shows that acetonitrile is less effective in the quantity of compound extracted with the peak areas being considerably lower. In positive ionization mode, acetonitrile shows the lowest peak areas for all compounds except *O*-acetylserine and 4-hydroxy-l-glutamate. Not unsurprisingly, the mixed solvent systems generally give the largest peak areas.

Average LogP values for each extraction (Supplementary Materials Table S3) indicated that acetonitrile extracted more nonpolar compounds than methanol-containing mixtures. The average LogP for the compounds extracted by acetonitrile is –0.92 while those extracted by methanol is –1.13 and the mixtures are –1.03 and –0.97 (MeCN:MeOH and MTBE:MeOH, respectively). This is not unexpected if the relative LogP values of the solvents themselves are compared as acetonitrile is less polar than methanol. This might account for the lower quantity of compounds extracted by acetonitrile. Acetonitrile favors the extraction of nonpolar compounds, and these have already been removed during the defatting process.

This study aimed to develop the tools required for consistent and reproducible analysis of the milk metabolome. It is just the first step and we are undertaking more extensive investigations. A limitation of the current study is that only one milk source was screened, and in future we aim to examine different sources, seasonal variations and raw milk versus processed. As we gain more data, we will develop in-house libraries that will help reduce the number of unannotated features, and we will explore the use of other solvents capture more of the chemical space.

## 5. Conclusions

After evaluating fifteen columns, covering both reverse phase and HILIC chemistries, we have concluded that a Waters ACQUITY Premier BEH Amide column provides a good compromise between retention time and separation across a cross section of appropriate compounds. This makes it ideal for untargeted metabolomic studies of cow’s milk samples. Four extraction solvents were investigated, i.e., acetonitrile, methanol, acetonitrile:methanol, and methyl *tert*-butyl ether:methanol, and it was determined that the importance of solvent selection reflects the aim of the study; when studying the differences between samples, the choice of solvent is important with each solvent covering a different volume of metabolite space. The wrong choice of solvent could easily lead to biased results or even missing key differences. Studies involving annotated compounds appear to be less sensitive to solvent selection, although we anticipate that this might change as spectral databases increase in size. The libraries of annotated compounds have been built using these common solvent choices, and the most common compounds have been confidently identified [49]; as the libraries expand and more esoteric molecules are confidently identified and added to the libraries, the differences will become apparent. As milk samples are commonly defatted before extraction, the use of acetonitrile is less effective as it favors nonpolar compounds that are frequently removed at this stage.

## Data Availability

The data presented in this study are available on request from the corresponding author due to commercial policy.

## Supplementary Materials

The following supporting information can be downloaded at https://www.mdpi.com/article/10.3390/metabo15090597/s1: Table S1: List of standards used; Table S2: Analytical Columns; Table S3: Compounds found in different milk extracts (POS mode) and average LogP; Table S4: Compounds found in different milk extracts (NEG mode).

## References

[1] Foroutan A., Guo A.C., Vazquez-Fresno R., Lipfert M., Zhang L., Zheng J., Badran H., Budinski Z., Mandal R., Ametaj B.N. (2019). Chemical Composition of Commercial Cow’s Milk. J. Agric. Food Chem..

[2] Ametaj B.N. (2020). Introducing Dairy: A Transdisciplinary Journal to Advance Understanding of Dairy Nutrition, Health and Productivity, Welfare and Well-Being as Well as Milk Synthesis-Composition and Health Effects of Its Products. Dairy.

[3] Visioli F., Strata A. (2014). Milk, Dairy Products, and Their Functional Effects in Humans: A Narrative Review of Recent Evidence. Adv. Nutr..

[4] Alothman M., Hogan S.A., Hennessy D., Dillon P., Kilcawley K.N., O’Donovan M., Tobin J., Fenelon M.A., O’Callaghan T.F. (2019). The “Grass-Fed” Milk Story: Understanding the Impact of Pasture Feeding on the Composition and Quality of Bovine Milk. Foods.

[5] Fiehn O. (2002). Metabolomics—The link between genotypes and phenotypes. Plant Mol. Biol..

[6] Guijas C., Montenegro-Burke J.R., Warth B., Spilker M.E., Siuzdak G. (2018). Metabolomics activity screening for identifying metabolites that modulate phenotype. Nat. Biotechnol..

[7] Johnson C.H., Ivanisevic J., Siuzdak G. (2016). Metabolomics: Beyond biomarkers and towards mechanisms. Nat. Rev. Mol. Cell Biol..

[8] Li Q., Yu Z., Zhu D., Meng X., Pang X., Liu Y., Frew R., Chen H., Chen G. (2017). The application of NMR-based milk metabolite analysis in milk authenticity identification. J. Sci. Food Agric..

[9] Loudiyi M., Temiz H.T., Sahar A., Haseeb Ahmad M., Boukria O., Hassoun A., Aït-Kaddour A. (2022). Spectroscopic techniques for monitoring changes in the quality of milk and other dairy products during processing and storage. Crit. Rev. Food Sci..

[10] Evans A.M., O’Donovan C., Playdon M., Beecher C., Beger R.D., Bowden J.A., Broadhurst D., Clish C.B., Dasari S., Dunn W.B. (2020). Dissemination and analysis of the quality assurance (QA) and quality control (QC) practices of LC–MS based untargeted metabolomics practitioners. Metabolomics.

[11] Raftery D. (2014). Mass Spectrometry in Metabolomics.

[12] Schrimpe-Rutledge A.C., Codreanu S.G., Sherrod S.D., McLean J.A. (2016). Untargeted Metabolomics Strategies—Challenges and Emerging Directions. J. Am. Soc. Mass Spectrom..

[13] Imperiale S., Morozova K., Ferrentino G., Scampicchio M. (2023). Analysis of milk with liquid chromatography–mass spectrometry: A review. Eur. Food Res. Technol..

[14] Yuan X., Shi W., Jiang J., Li Z., Fu P., Yang C., Rehman S.u., Pauciullo A., Liu Q., Shi D. (2022). Comparative metabolomics analysis of milk components between Italian Mediterranean buffaloes and Chinese Holstein cows based on LC-MS/MS technology. PLoS ONE.

[15] Suh J.H. (2022). Critical review: Metabolomics in dairy science—Evaluation of milk and milk product quality. Food Res. Int..

[16] Rocchetti G., Gallo A., Nocetti M., Lucini L., Masoero F. (2020). Milk metabolomics based on ultra-high-performance liquid chromatography coupled with quadrupole time-of-flight mass spectrometry to discriminate different cows feeding regimens. Food Res. Int..

[17] Barnes S., Benton H.P., Casazza K., Cooper S.J., Cui X., Du X., Engler J., Kabarowski J.H., Li S., Pathmasiri W. (2016). Training in metabolomics research. I. Designing the experiment, collecting and extracting samples and generating metabolomics data. J. Mass Spectrom..

[18] Liu X., Locasale J.W. (2017). Metabolomics: A Primer. Trends Biochem. Sci..

[19] Naz S., Vallejo M., García A., Barbas C. (2014). Method validation strategies involved in non-targeted metabolomics. J. Chromatogr. A.

[20] Garwolińska D., Namieśnik J., Kot-Wasik A., Hewelt-Belka W. (2019). State of the art in sample preparation for human breast milk metabolomics—Merits and limitations. TrAC Trends Anal. Chem..

[21] Li M., Kang S., Zheng Y., Shao J., Zhao H., An Y., Cao G., Li Q., Yue X., Yang M. (2020). Comparative metabolomics analysis of donkey colostrum and mature milk using ultra-high-performance liquid tandem chromatography quadrupole time-of-flight mass spectrometry. J. Dairy Sci..

[22] Zhan X. (2021). Metabolomics—Methodology and Applications in Medical Sciences and Life Sciences.

[23] Canelas A.B., ten Pierick A., Ras C., Seifar R.M., van Dam J.C., van Gulik W.M., Heijnen J.J. (2009). Quantitative Evaluation of Intracellular Metabolite Extraction Techniques for Yeast Metabolomics. Anal. Chem..

[24] Lin Y., Caldwell G.W., Li Y., Lang W., Masucci J. (2020). Inter-laboratory reproducibility of an untargeted metabolomics GC–MS assay for analysis of human plasma. Sci. Rep..

[25] Chiavelli L.U.R., Godoy A.C., Silveira R.d., Santos P.D.S., Lopes T.A.M., Santos O.O., Visentainer J.V. (2020). Optimization of Milk Sample Cleanup Using Response Surface Methodology. Food Anal. Methods.

[26] Hu C., Duan Q., Han X. (2020). Strategies to Improve/Eliminate the Limitations in Shotgun Lipidomics. Proteomics.

[27] Koivusalo M., Haimi P., Heikinheimo L., Kostiainen R., Somerharju P. (2001). Quantitative determination of phospholipid compositions by ESI-MS: Effects of acyl chain length, unsaturation, and lipid concentration on instrument response. J. Lipid Res..

[28] Ashokan M., Ramesha K.P., Hallur S., Karthikkeyan G., Rana E., Azharuddin N., Raj S.R., Jeyakumar S., Kumaresan A., Kataktalware M.A. (2021). Differences in milk metabolites in Malnad Gidda (Bos indicus) cows reared under pasture-based feeding system. Sci. Rep..

[29] Zhou B., Xiao J.F., Tuli L., Ressom H.W. (2012). LC-MS-based metabolomics. Mol. BioSyst..

[30] Wernisch S., Pennathur S. (2016). Evaluation of coverage, retention patterns, and selectivity of seven liquid chromatographic methods for metabolomics. Anal. Bioanal. Chem..

[31] Manis C., Scano P., Nudda A., Carta S., Pulina G., Caboni P. (2021). LC-QTOF/MS Untargeted Metabolomics of Sheep Milk under Cocoa Husks Enriched Diet. Dairy.

[32] Nobile M., Chiesa L.M., Danesi L., Fontana M., Ghidini S., Villa R.E., Panseri S. (2025). Metabolomic and volatilome profiling of milk to assess the application of infrared radiation processing. Food Control.

[33] Yan R., Ji Z., Fan J., Li J., Ren Y. (2024). Evaluation of the Efficacy of a Lactobacilli-Based Teat Detergents for the Microbiota of Cows Teats Using an Untargeted Metabolomics Approach. J. Microbiol. Biotechnol..

[34] Zhang Y.D., Li P., Zheng N., Jia Z.W., Meruva N., Ladak A., Cleland G., Wen F., Li S.L., Zhao S.G. (2018). A metabolomics approach to characterize raw, pasteurized, and ultra-high temperature milk using ultra-performance liquid chromatography—Quadrupole time-of-flight mass spectrometry and multivariate data analysis. J. Dairy Sci..

[35] Liu Z., Jiang A., Lv X., Zhou C., Tan Z. (2024). Metabolic Changes in Serum and Milk of Holstein Cows in Their First to Fourth Parity Revealed by Biochemical Analysis and Untargeted Metabolomics. Animals.

[36] Riboni N., Piergiovanni M., Mattarozzi M., Robotti E., Stocco G., Ablondi M., Cipolat-Gotet C., Summer A., Bianchi F., Careri M. (2025). Ultra-high performance liquid chromatography ion mobility-high-resolution mass spectrometry for the assessment of raw milk traceability. Food Chem..

[37] Sánchez M.C., Soria E., Llama-Palacios A., Almirón F., Valdés A., Cifuentes A., Hernández M., Ciudad M.J., Collado L. (2025). Lactic Microbiota and Metabolites in Raw Cow’s Milk: Implications for Consumer Health. Dairy.

[38] Ma S., Wang D., Zhang M., Xu L., Fu X., Zhang T., Yan M., Huang X. (2025). Untargeted metabonomic analysis reveals the composition and changes of milk metabolites in dual-purpose cattle (Bos taurus) population. J. Agric. Food Res..

[39] Zhuang J., Hou Y., Wang Y., Gao Y., Chen Y., Qi J., Li P., Bian Y., Ju N. (2024). Relationship between microorganisms and milk metabolites during quality changes in refrigerated raw milk: A metagenomic and metabolomic exploration. Int. J. Food Microbiol..

[40] Kenney D.H., Paffenroth R.C., Timko M.T., Teixeira A.R. (2023). Dimensionally reduced machine learning model for predicting single component octanol–water partition coefficients. J. Cheminform..

[41] Tan D., Zhang X., Su M., Jia M., Zhu D., Kebede B., Wu H., Chen G. (2021). Establishing an untargeted-to-MRM liquid chromatography–mass spectrometry method for discriminating reconstituted milk from ultra-high temperature milk. Food Chem..

[42] Broadhurst D., Goodacre R., Reinke S.N., Kuligowski J., Wilson I.D., Lewis M.R., Dunn W.B. (2018). Guidelines and considerations for the use of system suitability and quality control samples in mass spectrometry assays applied in untargeted clinical metabolomic studies. Metabolomics.

[43] Patil I. (2021). Visualizations with statistical details: The ’ggstatsplot’ approach. J. Open Source Softw..

[44] Chen H., Boutros P.C. (2011). VennDiagram: A package for the generation of highly-customizable Venn and Euler diagrams in R. BMC Bioinform..

[45] Lewis M.R., Pearce J.T.M., Spagou K., Green M., Dona A.C., Yuen A.H.Y., David M., Berry D.J., Chappell K., Horneffer-van der Sluis V. (2016). Development and Application of Ultra-Performance Liquid Chromatography-TOF MS for Precision Large Scale Urinary Metabolic Phenotyping. Anal. Chem..

[46] Want E.J., Wilson I.D., Gika H., Theodoridis G., Plumb R.S., Shockcor J., Holmes E., Nicholson J.K. (2010). Global metabolic profiling procedures for urine using UPLC-MS. Nat. Protoc..

[47] Broeckling C.D., Beger R.D., Cheng L.L., Cumeras R., Cuthbertson D.J., Dasari S., Davis W.C., Dunn W.B., Evans A.M., Fernández-Ochoa A. (2023). Current Practices in LC-MS Untargeted Metabolomics: A Scoping Review on the Use of Pooled Quality Control Samples. Anal. Chem..

[48] Dudzik D., Barbas-Bernardos C., García A., Barbas C. (2018). Quality assurance procedures for mass spectrometry untargeted metabolomics. a review. J. Pharm. Biomed. Anal..

[49] Schymanski E.L., Jeon J., Gulde R., Fenner K., Ruff M., Singer H.P., Hollender J. (2014). Identifying Small Molecules via High Resolution Mass Spectrometry: Communicating Confidence. Environ. Sci. Technol..

