# Supplementary material for: Methods for Untargeted Analysis of Milk Metabolites: Influence of Extraction Method and Optimization of Separation
Source: Metabolites. 2025 Sep 8;15(9):597. doi: 10.3390/metabo15090597 (PMC12471616; doi:10.3390/metabo15090597)
Supplement: Supplementary file 1 [file metabolites-15-00597-s001.zip › 02_SupplementaryData.pdf]

Table S1 – list of standards used

Compound	Supplier
Alanine	Sigma Aldrich (Saint Louis, MO, USA)
Allantoin	Sigma Aldrich (Saint Louis, MO, USA)
Arginine	Sigma Aldrich (Saint Louis, MO, USA)
Ascorbic Acid	Sigma Aldrich (Saint Louis, MO, USA)
Aspartic Acid	Sigma Aldrich (Saint Louis, MO, USA)
Benzoic Acid	Sigma Aldrich (Saint Louis, MO, USA)
Biuret	Sigma Aldrich (Saint Louis, MO, USA)
Caffeic Acid	Sigma Aldrich (Saint Louis, MO, USA)
Caffeine	Sigma Aldrich (Saint Louis, MO, USA)
Cystine	Sigma Aldrich (Saint Louis, MO, USA)
Glutamic Acid	Sigma Aldrich (Saint Louis, MO, USA)
Glycine	Sigma Aldrich (Saint Louis, MO, USA)
Hippuric Acid	Sigma Aldrich (Saint Louis, MO, USA)
Histidine	Sigma Aldrich (Saint Louis, MO, USA)
Isoleucine	Sigma Aldrich (Saint Louis, MO, USA)
Leucine	Sigma Aldrich (Saint Louis, MO, USA)
Lysine	Sigma Aldrich (Saint Louis, MO, USA)
Methionine	Sigma Aldrich (Saint Louis, MO, USA)
Nicotinamide	Sigma Aldrich (Saint Louis, MO, USA)
Nicotinic Acid	Sigma Aldrich (Saint Louis, MO, USA)
Palmitic Acid	Sigma Aldrich (Saint Louis, MO, USA)
Phenylalanine	Sigma Aldrich (Saint Louis, MO, USA)
Proline	Sigma Aldrich (Saint Louis, MO, USA)

Serine	Sigma Aldrich (Saint Louis, MO, USA)
Threonine	Sigma Aldrich (Saint Louis, MO, USA)
Tryptophan	Sigma Aldrich (Saint Louis, MO, USA)
Tyrosine	Sigma Aldrich (Saint Louis, MO, USA)
Uric Acid	Sigma Aldrich (Saint Louis, MO, USA)
Valine	Sigma Aldrich (Saint Louis, MO, USA)
Xanthine	Sigma Aldrich (Saint Louis, MO, USA)
8-Bromocaffeine	Sigma Aldrich (Saint Louis, MO, USA)
Choline	Sigma Aldrich (Saint Louis, MO, USA)
Hypoxanthine	Sigma Aldrich (Saint Louis, MO, USA)
Uracil	Sigma Aldrich (Saint Louis, MO, USA)

Table S2 – Analytical Columns

Column	Specifications	Manufacturer Part Number
Sigma-Aldrich Ascentis Express HILIC	150 mm, 2.1 mm, 2.7 µm	53946-U
Phenomenex Kinetex HILIC	150 mm, 2.1 mm, 2.6 µm	00F-4461-AN
Phenomenex Luna HILIC	150 mm, 2.0 mm, 3 µm	00F-4449-B0
Waters ACQUITY Premier BEH amide	150 mm, 2.1 mm, 1.7 µm	186004802
Shodex HILIC	150 mm, 2.0 mm, 5 µm	F7600006
SeQuant ZIC-HILIC	150 mm, 2.1 mm, 3.5 µm	1.50442.0001
biozen Glycan	150 mm, 2.1 mm, 2.6 µm	00FF-4773-AN
Accucore HILIC	150 mm, 2.1 mm, 2.6 µm	THC17526
Phenomenex Gemini C18	150 mm, 2.0 mm, 3.0 µm	00F-4439-B0
Phenomenex Kinetex F5	150 mm, 2.1 mm, 1.7 µm	00F-4722-AN
Phenomenex Luna Omega C18 Polar	150 mm, 2.1 mm, 1.6 µm	00F-4748-AN
Phenomenex Kinetex XB-C18	150 mm, 2.1 mm, 1.7 µm	00F-4498-AN
Phenomenex Kinetex Biphenyl	150 mm, 2.1 mm, 1.7 µm	00F-4628-AN
Phenomenex Kinetex C18	150 mm, 2.1 mm, 1.7 µm	00F-4475-AN
Phenomenex Luna Omega C18	150 mm, 2.1 mm, 1.6 µm	00F-4742-AN

Table S3 – Compounds found in different milk extracts (POS mode) and average LogP

**MeCN**	**MeOH**	**MeCN&MeOH**	**MTBE&MeOH**	**LogP**

L-Carnitine	L-Carnitine	L-Carnitine	L-Carnitine	-0.2
1,2-Benzoquinone	1,2-Benzoquinone	1,2-Benzoquinone	1,2-Benzoquinone	0.4
Choline	Choline	Choline	Choline	-0.4
1,2-diacylglycerol	1,2-diacylglycerol	1,2-diacylglycerol	1,2-diacylglycerol	-0.3
Glycerophosphocholine	Glycerophosphocholine	Glycerophosphocholine	Glycerophosphocholine	-2.3
1-Methyladenosine	1-Methyladenosine	1-Methyladenosine	1-Methyladenosine	-1
DL-2-Aminooctanoic acid	DL-2-Aminooctanoic acid	DL-2-Aminooctanoic acid	DL-2-Aminooctanoic acid	-0.5
1-Methylhistidine	1-Methylhistidine	1-Methylhistidine	1-Methylhistidine	-3.3
L-Acetylcarnitine	L-Acetylcarnitine	L-Acetylcarnitine	L-Acetylcarnitine	0.4
2-Ethylacryloylcarnitine	2-Ethylacryloylcarnitine	2-Ethylacryloylcarnitine	2-Ethylacryloylcarnitine	1.8
D-Maltose	D-Maltose	D-Maltose	D-Maltose	-4.7
2-Heptanone	Not Found	Not Found	Not Found	1.98
Urea	Urea	Urea	Urea	-2.11
2-Indolecarboxylic acid	2-Indolecarboxylic acid	2-Indolecarboxylic acid	2-Indolecarboxylic acid	2.31
Creatine	Creatine	Creatine	Creatine	-1.2
2-Methylbutyroylcarnitine	2-Methylbutyroylcarnitine	2-Methylbutyroylcarnitine	2-Methylbutyroylcarnitine	1.8
2-Octenedioic acid	2-Octenedioic acid	2-Octenedioic acid	2-Octenedioic acid	0.9
2-Phenylglycine	Not Found	Not Found	Not Found	-2.07
α-Lactose	α-Lactose	α-Lactose	α-Lactose	-4.7
4-Hydroxyphenylpyruvic acid	4-Hydroxyphenylpyruvic acid	4-Hydroxyphenylpyruvic acid	Not Found	0.9
Isobutyryl-L-carnitine	Isobutyryl-L-carnitine	Isobutyryl-L-carnitine	Isobutyryl-L-carnitine	1.4
5-(2-Hydroxyethyl)-4-methylthiazole	5-(2-Hydroxyethyl)-4-methylthiazole	5-(2-Hydroxyethyl)-4-methylthiazole	5-(2-Hydroxyethyl)-4-methylthiazole	0.8
Piperidine	Piperidine	Piperidine	Piperidine	0.84
5-(6-hydroxy-6-methyloctyl)-2,5-dihydrofuran-2-one	Not Found	Not Found	Not Found	Not Found
Propionylcarnitine	Propionylcarnitine	Propionylcarnitine	Propionylcarnitine	0.9
5-hydroxy-4-methoxy-5,6-dihydro-2H-pyran-2-one	5-hydroxy-4-methoxy-5,6-dihydro-2H-pyran-2-one	Not Found	5-hydroxy-4-methoxy-5,6-dihydro-2H-pyran-2-one	-0.7
Acetylcholine	Acetylcholine	Acetylcholine	Acetylcholine	0.2
5-Hydroxy-L-tryptophan	5-Hydroxy-L-tryptophan	5-Hydroxy-L-tryptophan	5-Hydroxy-L-tryptophan	-2.05
5-Methoxyindoleacetate	5-Methoxyindoleacetate	5-Methoxyindoleacetate	5-Methoxyindoleacetate	1.4
Pantothenic acid	Pantothenic acid	Pantothenic acid	Pantothenic acid	-1.1
5-Methylcytosine	5-Methylcytosine	5-Methylcytosine	5-Methylcytosine	-0.8

Betaine	Betaine	Betaine	Betaine	0.5
7-Methylguanine	7-Methylguanine	7-Methylguanine	7-Methylguanine	-1.1
Creatinine	Creatinine	Creatinine	Creatinine	-1.8
Acetoacetic acid	Acetoacetic acid	Acetoacetic acid	Acetoacetic acid	-0.4
L-Lactic acid	L-Lactic acid	L-Lactic acid	L-Lactic acid	-0.7
Benzylamine	Not Found	Not Found	Not Found	1.1
L-Glutamic acid	L-Glutamic acid	L-Glutamic acid	L-Glutamic acid	-3.69
Trimethylamine N-oxide	Trimethylamine N-oxide	Trimethylamine N-oxide	Trimethylamine N-oxide	-2.57
Biotin	Biotin	Biotin	Biotin	0.5
Pyridoxal	Pyridoxal	Pyridoxal	Pyridoxal	0
Butyric acid	Not Found	Butyric acid	Butyric acid	0.8
Caprylic acid	Caprylic acid	Caprylic acid	Caprylic acid	3.05
Hydroxybutyrylcarnitine	Hydroxybutyrylcarnitine	Hydroxybutyrylcarnitine	Hydroxybutyrylcarnitine	0.1
Glucosamine 6-phosphate	Glucosamine 6-phosphate	Glucosamine 6-phosphate	Glucosamine 6-phosphate	-6.8
Phosphocreatine	Phosphocreatine	Phosphocreatine	Phosphocreatine	2.6
Cyclic AMP	Cyclic AMP	Cyclic AMP	Cyclic AMP	-2.96
Thiamine	Thiamine	Thiamine	Thiamine	1
Cytidine	Cytidine	Cytidine	Cytidine	-2.51
Diethanolamine	Diethanolamine	Diethanolamine	Diethanolamine	-1.4
Cytidine monophosphate	Cytidine monophosphate	Cytidine monophosphate	Cytidine monophosphate	-3.4
p-Aminobenzoic acid	p-Aminobenzoic acid	p-Aminobenzoic acid	p-Aminobenzoic acid	0.83
Cytosine	Cytosine	Cytosine	Cytosine	-1.73
Not Found	Trigonelline	Not Found	Not Found	1.2
Not Found	N-(2-hydroxyphenyl)acetamide	N-(2-hydroxyphenyl)acetamide	N-(2-hydroxyphenyl)acetamide	0.7
Not Found	p-Cresol	p-Cresol	p-Cresol	-2.31
Not Found	Pyridoxamine	Pyridoxamine	Not Found	-1
Not Found	Pipecolic acid	Not Found	Pipecolic acid	-2.31
Not Found	5'-Methylthioadenosine	5'-Methylthioadenosine	Not Found	-0.3
Not Found	L-Threonine	L-Threonine	L-Threonine	-2.94
Not Found	Pro-Leu-Lys	Not Found	Pro-Leu-Lys	-3.2
Not Found	Dopamine	Not Found	Dopamine	-0.98
Not Found	Adenosine monophosphate	Adenosine monophosphate	Adenosine monophosphate	-3.1
Not Found	L-Serine	L-Serine	L-Serine	-3.07
Not Found	Not Found	3-Aminopropionaldehyde	Not Found	-1.3
Not Found	Not Found	Arecoline	Not Found	0.35

Not Found	Not Found	5-(6-Methyl-7-hydroxyoctyl)furan-2(5H)-one	Not Found	Not Found
Not Found	Not Found	L-Aspartic acid	Not Found	-3.89
Not Found	Not Found	Hydroxyproline	Hydroxyproline	-3.17
Not Found	Not Found	Phosphocreatinine	Not Found	-2.2
Not Found	Not Found	4-Hydroxycinnamic acid	4-Hydroxycinnamic acid	1.79
Not Found	Not Found	(9Z)-3-Hydroxyoctadecenoylcarnitine	Not Found	6.5
Not Found	Not Found	5-Tetradecenoic acid	Not Found	5.1
Not Found	Not Found	(E,E)-2,4-Hexadienal	(E,E)-2,4-Hexadienal	1.2
Not Found	Not Found	Not Found	Allysine	-3.2
Not Found	Not Found	Not Found	methyl 2-(1,3,5-trihydroxy-4a-methyl-8-oxo-decahydronaphthalen-2-yl)prop-2-enoate	Not Found
Not Found	Not Found	Not Found	L-Octanoylcarnitine	3.4
Not Found	Not Found	Not Found	11-Dodecenoic acid	4.4
Not Found	Not Found	Not Found	N-Hydroxy-l-tyrosine	-1.7
D-Glucose	D-Glucose	D-Glucose	D-Glucose	-2.6
Fructose 6-phosphate	Fructose 6-phosphate	Fructose 6-phosphate	Fructose 6-phosphate	-4.3
L-Alanine	L-Alanine	L-Alanine	L-Alanine	-2.85
DL-Stachydrine	DL-Stachydrine	DL-Stachydrine	Not Found	0.4
Deoxycytidine	Deoxycytidine	Deoxycytidine	Deoxycytidine	-1.8
Hippuric acid	Hippuric acid	Hippuric acid	Hippuric acid	0.31
Diacetyl	Diacetyl	Diacetyl	Diacetyl	-1.3
2-Methyl-3-[3,4,5-trihydroxy-6-(hydroxymethyl)tetrahydro-2H-pyran-2-yloxy]-4H-pyran-4-one	2-Methyl-3-[3,4,5-trihydroxy-6-(hydroxymethyl)tetrahydro-2H-pyran-2-yloxy]-4H-pyran-4-one	2-Methyl-3-[3,4,5-trihydroxy-6-(hydroxymethyl)tetrahydro-2H-pyran-2-yloxy]-4H-pyran-4-one	2-Methyl-3-[3,4,5-trihydroxy-6-(hydroxymethyl)tetrahydro-2H-pyran-2-yloxy]-4H-pyran-4-one	Not Found
L-Proline	L-Proline	L-Proline	L-Proline	-2.54
Dimethyl-L-arginine	Dimethyl-L-arginine	Dimethyl-L-arginine	Dimethyl-L-arginine	-3.6
Trimethylamine	Trimethylamine	Trimethylamine	Trimethylamine	0.16
L-Arginine	L-Arginine	L-Arginine	L-Arginine	-4.2
Docosahexaenoic acid	Not Found	Not Found	Not Found	6.2
L-Methionine	L-Methionine	L-Methionine	L-Methionine	-1.87
Ethanolamine	Ethanolamine	Ethanolamine	Ethanolamine	-1.31
Nicotinamide	Nicotinamide	Nicotinamide	Nicotinamide	-0.4
N6,N6,N6-Trimethyl-L-lysine	N6,N6,N6-Trimethyl-L-lysine	N6,N6,N6-Trimethyl-L-lysine	N6,N6,N6-Trimethyl-L-lysine	-1.6

Glycerol 3-phosphate	Glycerol 3-phosphate	Glycerol 3-phosphate	Glycerol 3-phosphate	-2.9
Glycine	Glycine	Glycine	Glycine	-3.2
L-Glutamine	L-Glutamine	L-Glutamine	L-Glutamine	-3.64
Guanine	Guanine	Guanine	Guanine	-0.91
Pyroglutamic acid	Pyroglutamic acid	Pyroglutamic acid	Pyroglutamic acid	-0.8
Hexanoylcarnitine	Hexanoylcarnitine	Hexanoylcarnitine	Hexanoylcarnitine	2.3
Riboflavin	Riboflavin	Riboflavin	Riboflavin	-1.46
L-Isoleucine	L-Isoleucine	L-Isoleucine	L-Isoleucine	-1.7
N-Acetylneuraminic acid	N-Acetylneuraminic acid	N-Acetylneuraminic acid	N-Acetylneuraminic acid	-3.5
Hypoxanthine	Hypoxanthine	Hypoxanthine	Hypoxanthine	-1.11
Indolylacryloylglycine	Indolylacryloylglycine	Indolylacryloylglycine	Indolylacryloylglycine	1.3
Phosphoric acid	Phosphoric acid	Phosphoric acid	Phosphoric acid	-2.15
Inosine	Inosine	Inosine	Inosine	-2.1
Methylmalonylcarnitine	Methylmalonylcarnitine	Methylmalonylcarnitine	Methylmalonylcarnitine	0.6
Isopropyl beta-D-glucoside	Isopropyl beta-D-glucoside	Isopropyl beta-D-glucoside	Not Found	-1.4
N-Acetylgalactosamine	N-Acetylgalactosamine	N-Acetylgalactosamine	N-Acetylgalactosamine	-1.7
Isovaleric acid	Not Found	Not Found	Not Found	1.16
N-Acetyl-D-lactosamine	N-Acetyl-D-lactosamine	N-Acetyl-D-lactosamine	N-Acetyl-D-lactosamine	-4.7
L-Asparagine	L-Asparagine	L-Asparagine	L-Asparagine	-3.82
Leucyl-Alanine	Leucyl-Alanine	Leucyl-Alanine	Leucyl-Alanine	-2.13
Taurine	Taurine	Taurine	Taurine	-4.1
Xestoaminol C	Xestoaminol C	Xestoaminol C	Xestoaminol C	4.7
Phenylacetylglycine	Phenylacetylglycine	Phenylacetylglycine	Phenylacetylglycine	0.7
L-Histidine	L-Histidine	L-Histidine	L-Histidine	-3.32
L-isoleucyl-L-proline	L-isoleucyl-L-proline	L-isoleucyl-L-proline	L-isoleucyl-L-proline	-1.8
L-Phenylalanine	L-Phenylalanine	L-Phenylalanine	L-Phenylalanine	-1.38
L-Tyrosine	L-Tyrosine	L-Tyrosine	L-Tyrosine	-2.26
Melezitose	Melezitose	Melezitose	Melezitose	-5.8
N-Heptanoylglycine	N-Heptanoylglycine	N-Heptanoylglycine	N-Heptanoylglycine	1.6
Ornithine	Ornithine	Ornithine	Ornithine	-4.22
Palmitoleic acid	Not Found	Not Found	Not Found	6.4
Phenyl-Leucine	Phenyl-Leucine	Phenyl-Leucine	Phenyl-Leucine	3.2
LysoPC(16:0)	LysoPC(16:0)	LysoPC(16:0)	LysoPC(16:0)	5.6
Pretyrosine	Not Found	Not Found	Not Found	-3.1
Toluene	Not Found	Toluene	Not Found	2.73
Leu-Pro-Lys	Not Found	Leu-Pro-Lys	Not Found	-3.1

Table S4 – Compounds found in different milk extracts (NEG mode)

**MeCN**	**MeOH**	**MeCN&MeOH**	**MTBE&MeOH**
Lactose	Lactose	Lactose	Lactose
Hippuric acid	Hippuric acid	Not Found	Hippuric acid
1,2-diacylglycerol	1,2-diacylglycerol	1,2-diacylglycerol	1,2-diacylglycerol
Oxalacetic acid	Oxalacetic acid	Oxalacetic acid	Oxalacetic acid
Erythrose	Erythrose	Erythrose	Erythrose
D-Glucose	D-Glucose	D-Glucose	D-Glucose
Phenylacetylglycine	Phenylacetylglycine	Not Found	Phenylacetylglycine
Citric acid	Citric acid	Citric acid	Citric acid
L-Lactic acid	L-Lactic acid	L-Lactic acid	L-Lactic acid
Acetoacetic acid	Acetoacetic acid	Acetoacetic acid	Acetoacetic acid
Propionic acid	Propionic acid	Propionic acid	Propionic acid
Glycine	Glycine	Not Found	Not Found
Citraconic acid	Citraconic acid	Citraconic acid	Citraconic acid
3-Furoic acid	3-Furoic acid	3-Furoic acid	3-Furoic acid
Acrylic acid	Acrylic acid	Acrylic acid	Acrylic acid
4-Hydroxybenzoic acid	4-Hydroxybenzoic acid	4-Hydroxybenzoic acid	4-Hydroxybenzoic acid
cis-Aconitic acid	cis-Aconitic acid	cis-Aconitic acid	cis-Aconitic acid
Acetic acid	Acetic acid	Acetic acid	Acetic acid
5-Methoxyindoleacetate	5-Methoxyindoleacetate	5-Methoxyindoleacetate	5-Methoxyindoleacetate
Pyruvic acid	Pyruvic acid	Pyruvic acid	Pyruvic acid
D-2-Hydroxyglutaric acid	D-2-Hydroxyglutaric acid	D-2-Hydroxyglutaric acid	D-2-Hydroxyglutaric acid
3-Indoxyl sulphate	3-Indoxyl sulphate	3-Indoxyl sulphate	3-Indoxyl sulphate
Pyridoxal	Pyridoxal	Pyridoxal	Pyridoxal
N-Acetylneuraminic acid	N-Acetylneuraminic acid	N-Acetylneuraminic acid	N-Acetylneuraminic acid
Oxoglutaric acid	Oxoglutaric acid	Oxoglutaric acid	Oxoglutaric acid
2-Hydroxybutyric acid	2-Hydroxybutyric acid	2-Hydroxybutyric acid	2-Hydroxybutyric acid
Salicyluric acid	Salicyluric acid	Salicyluric acid	Salicyluric acid
2-Pyrocatechuic acid	2-Pyrocatechuic acid	2-Pyrocatechuic acid	2-Pyrocatechuic acid
Gluconic acid	Gluconic acid	Gluconic acid	Gluconic acid
L-Malic acid	L-Malic acid	L-Malic acid	L-Malic acid
Uracil	Uracil	Uracil	Uracil
Fructose 6-phosphate	Fructose 6-phosphate	Fructose 6-phosphate	Fructose 6-phosphate
2-Methylcitric acid	2-Methylcitric acid	2-Methylcitric acid	2-Methylcitric acid
p-Cresol	p-Cresol	p-Cresol	p-Cresol
Benzoic acid	Benzoic acid	Benzoic acid	Benzoic acid
Phenol	Phenol	Phenol	Phenol
D-Ribulose 5-phosphate	D-Ribulose 5-phosphate	D-Ribulose 5-phosphate	D-Ribulose 5-phosphate
Riboflavin	Riboflavin	Riboflavin	Riboflavin

Cholic acid	Cholic acid	Cholic acid	Cholic acid
2-Phenylglycine	2-Phenylglycine	2-Phenylglycine	2-Phenylglycine
Homovanillic acid	Not Found	Homovanillic acid	Not Found
D-Ribose	D-Ribose	D-Ribose	D-Ribose
L-Acetylcarnitine	L-Acetylcarnitine	L-Acetylcarnitine	L-Acetylcarnitine
Fumaric acid	Fumaric acid	Fumaric acid	Fumaric acid
L-Histidine	L-Histidine	L-Histidine	L-Histidine
Deoxycytidine	Not Found	Not Found	Not Found
L-Fucose	L-Fucose	L-Fucose	L-Fucose
2-Phosphoglyceric acid	Not Found	Not Found	Not Found
(+/-)9,10-dihydroxy-12Z-octadecenoic acid	(+/-)9,10-dihydroxy-12Z-octadecenoic acid	(+/-)9,10-dihydroxy-12Z-octadecenoic acid	Not Found
Ribothymidine	Ribothymidine	Ribothymidine	Ribothymidine
1-Methylguanosine	1-Methylguanosine	1-Methylguanosine	1-Methylguanosine
p-Cresyl sulfate	p-Cresyl sulfate	p-Cresyl sulfate	p-Cresyl sulfate
Guanosine	Guanosine	Guanosine	Guanosine
Glycocholic acid	Glycocholic acid	Glycocholic acid	Glycocholic acid
D-Arabitol	D-Arabitol	D-Arabitol	D-Arabitol
Threoninyl-Aspartate	Threoninyl-Aspartate	Threoninyl-Aspartate	Threoninyl-Aspartate
Deoxycholic acid	Not Found	Not Found	Deoxycholic acid
2-Methylbutyroylcarnitine	2-Methylbutyroylcarnitine	2-Methylbutyroylcarnitine	2-Methylbutyroylcarnitine
4-Ethylphenol	4-Ethylphenol	4-Ethylphenol	4-Ethylphenol
Not Found	D-(+)-Galactose	Not Found	Not Found
Not Found	δ-Gluconic acid δ-lactone	Not Found	Not Found
Not Found	Diacetyl	Not Found	Not Found
Not Found	Epinephrine	Not Found	Not Found
Not Found	Phosphoric acid	Phosphoric acid	Phosphoric acid
Not Found	N-Acetylvaline	Not Found	Not Found
Not Found	Pantothenic acid	Not Found	Pantothenic acid
Not Found	Acetone	Not Found	Not Found
Not Found	L-(+)-Tartaric acid	L-(+)-Tartaric acid	L-(+)-Tartaric acid
Not Found	7-Methylxanthine	7-Methylxanthine	7-Methylxanthine
Not Found	Oleic acid	Oleic acid	Not Found
Not Found	Glycerol 3-phosphate	Glycerol 3-phosphate	Not Found
Not Found	Not Found	L-Tyrosine	L-Tyrosine
Not Found	Not Found	Inosine	Not Found
Not Found	Not Found	Ascorbic acid	Ascorbic acid
Not Found	Not Found	Mevalonolactone	Not Found
Not Found	Not Found	3-Methoxyphenylacetic acid	Not Found
Not Found	Not Found	Orotidine	Not Found

Not Found	Not Found	4-Hydroxyphenylpyruvic acid	Not Found
Not Found	Not Found	Tyrosyl-Leucine	Not Found
Not Found	Not Found	4-Aminophenol	4-Aminophenol
Not Found	Not Found	Not Found	12,13-dihydroxy-9Z-octadecenoic acid
Not Found	Not Found	Not Found	Dihydrothymine
Not Found	Not Found	Not Found	2-Keto-glutaramic acid
